# Supplementary material for: Mono-ubiquitination of Rabphilin 3A by UBE3A serves a non-degradative function
Source: Sci Rep. 2021 Feb 4;11:3007. doi: 10.1038/s41598-021-82319-9 (PMC7862399; doi:10.1038/s41598-021-82319-9)
Supplement: Supplementary file 1 — Supplementary Information. [file 41598_2021_82319_MOESM1_ESM.pdf]

Mono-ubiquitination of Rabphilin 3A by UBE3A serves a non-degradative function

Authors/Affiliations

Rossella Avagliano Trezza[1,2,5], A. Mattijs Punt[2,5], Edwin Mientjes[2], Marlene van den Berg[1], F. Isabella Zampeta[2], Ilona J. de Graaf[1,2], Yana van der Weegen[1], Jeroen A. A. Demmers[3], Ype Elgersma[2,4*], Ben Distel[1,2,4*]

[1]Department of Medical Biochemistry, Amsterdam UMC, University of Amsterdam, Amsterdam, 1105 AZ, The Netherlands

[2]Department of Neuroscience, Erasmus MC, Rotterdam, Rotterdam, 3015 CN, The Netherlands

[3]Proteomics Center, Erasmus MC, Rotterdam, 3015 CN, The Netherlands

[4]*ENCORE* Expertise Center for Neurodevelopmental Disorders, Erasmus MC, Rotterdam, Rotterdam, 3015 CN, The Netherlands

[5]These authors contributed equally

* Correspondence: b.distel@amsterdamumc.nl; y.elgersma@erasmusmc.nl

Supplementary Fig. 1

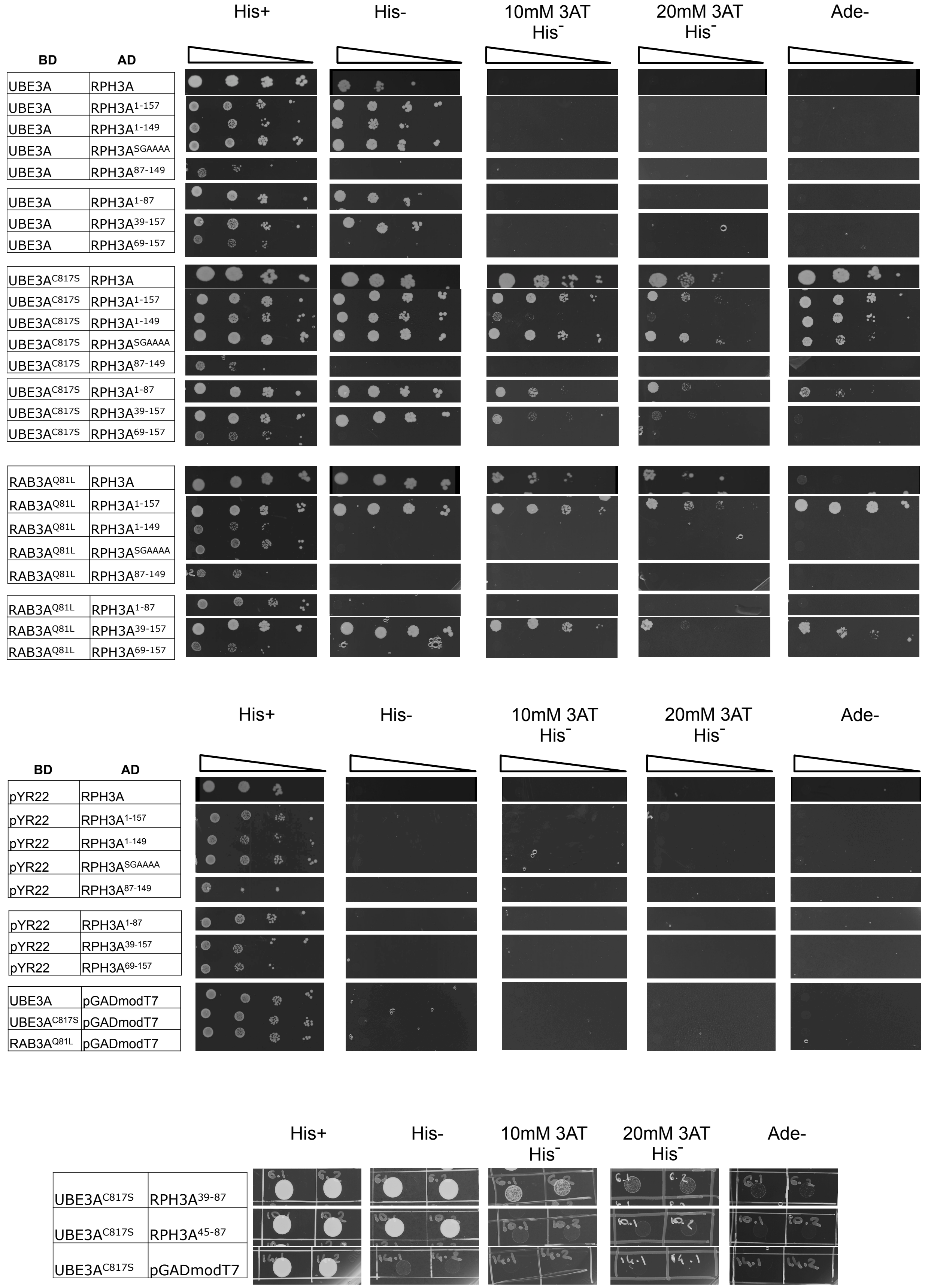

Supplementary Figure S1. Identification of RPH3A as a binding partner of UBE3A. Original plates of the spot assays corresponding to Figure 1.

Supplementary Fig. 2

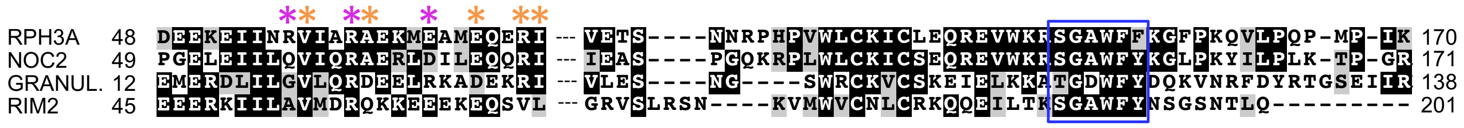

Supplementary Figure S2. Alignment of GTPase effectors. Multiple sequence alignment of different GTPase effectors: RPH3A, NOC2, GRANULOPHILLIN (GRANUL.) and RIM2. Two different regions of the N-terminus are shown: α-helix a1 (residues 48-71 in RPH3A) and the region C-terminal of the Zn-finger including the SGAWFF motif (residues124-170 in RPH3A). Residues that are identical in at least two proteins are shaded black, while similar residues are shaded grey. Asterisks mark residues that affect only the UBE3A interaction (magenta) or both UBE3A and RAB3A binding (orange). The blue box highlights the highly conserved SGAWFF domain.

Supplementary Fig. 3

A

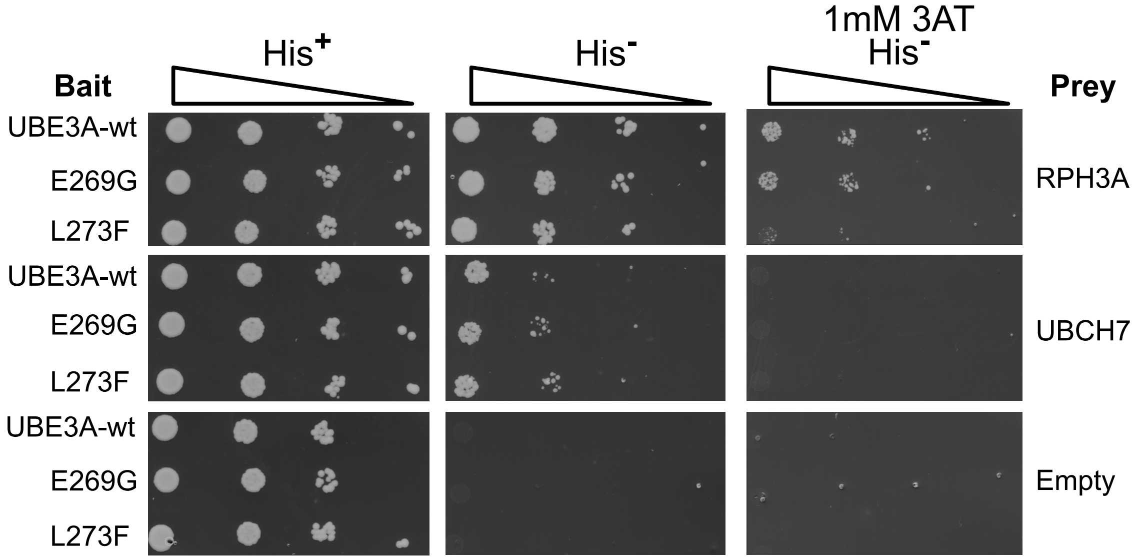

B

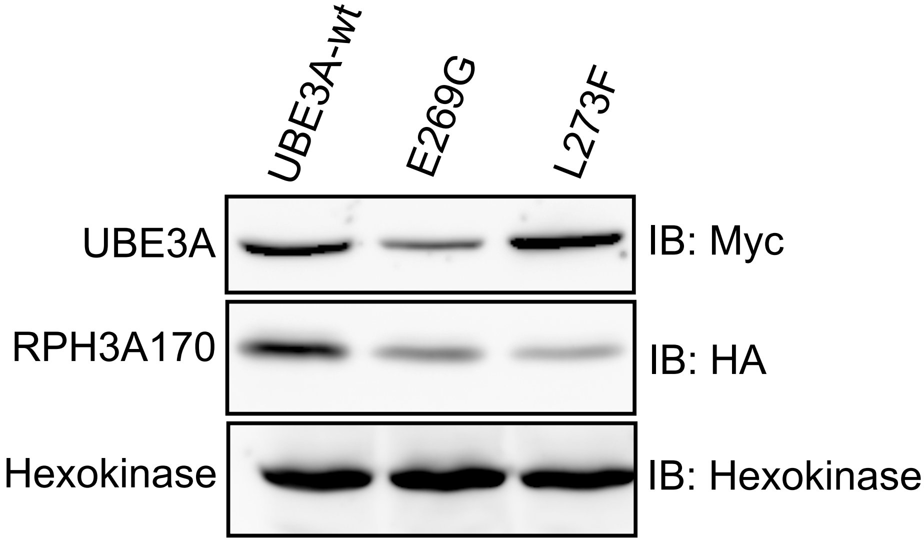

.

Supplementary Figure S3. An AS-linked mutation in the RPH3A binding site of UBE3A interferes with RPH3A binding. a) Original plates of the spot assay corresponding to **Figure 2c. b**) Equal expression of UBE3A variants. Total lysates obtained from yeast cells co-expressing the indicated Y2H constructs were analyzed by SDS-PAGE and immunoblotting using anti-myc (UBE3A) and anti-HA (RPH3A) antibodies. Hexokinase was used as loading control. Full-length blots are presented in **Supplementary Figure S12**

Supplementary Fig. 4

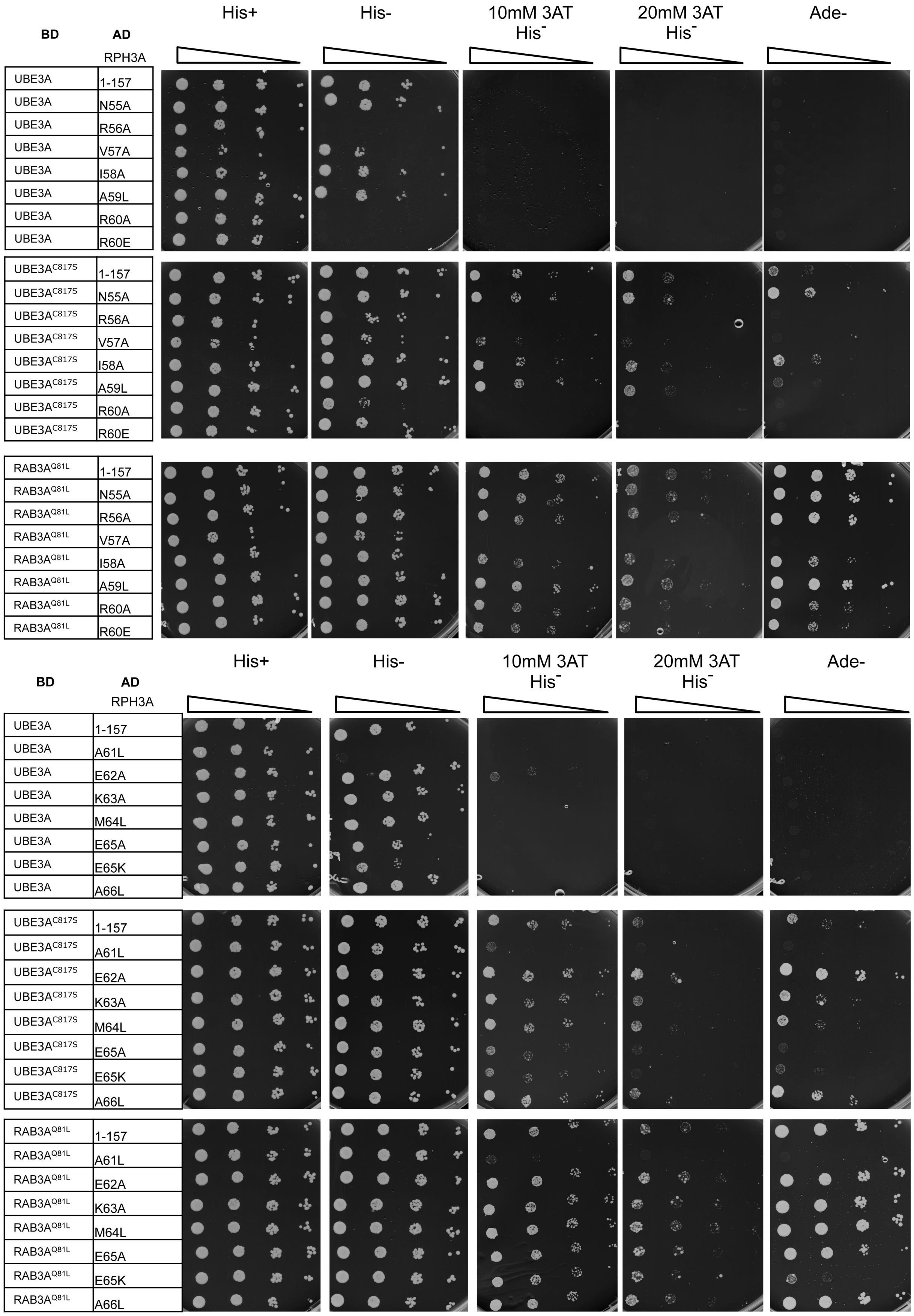

Supplementary Figure S4. Identification of the RPH3A residues involved in UBE3A and RAB3A interaction. Original plates of the spot assays corresponding to **Figure 3a**.

Supplementary Fig. 4 continued

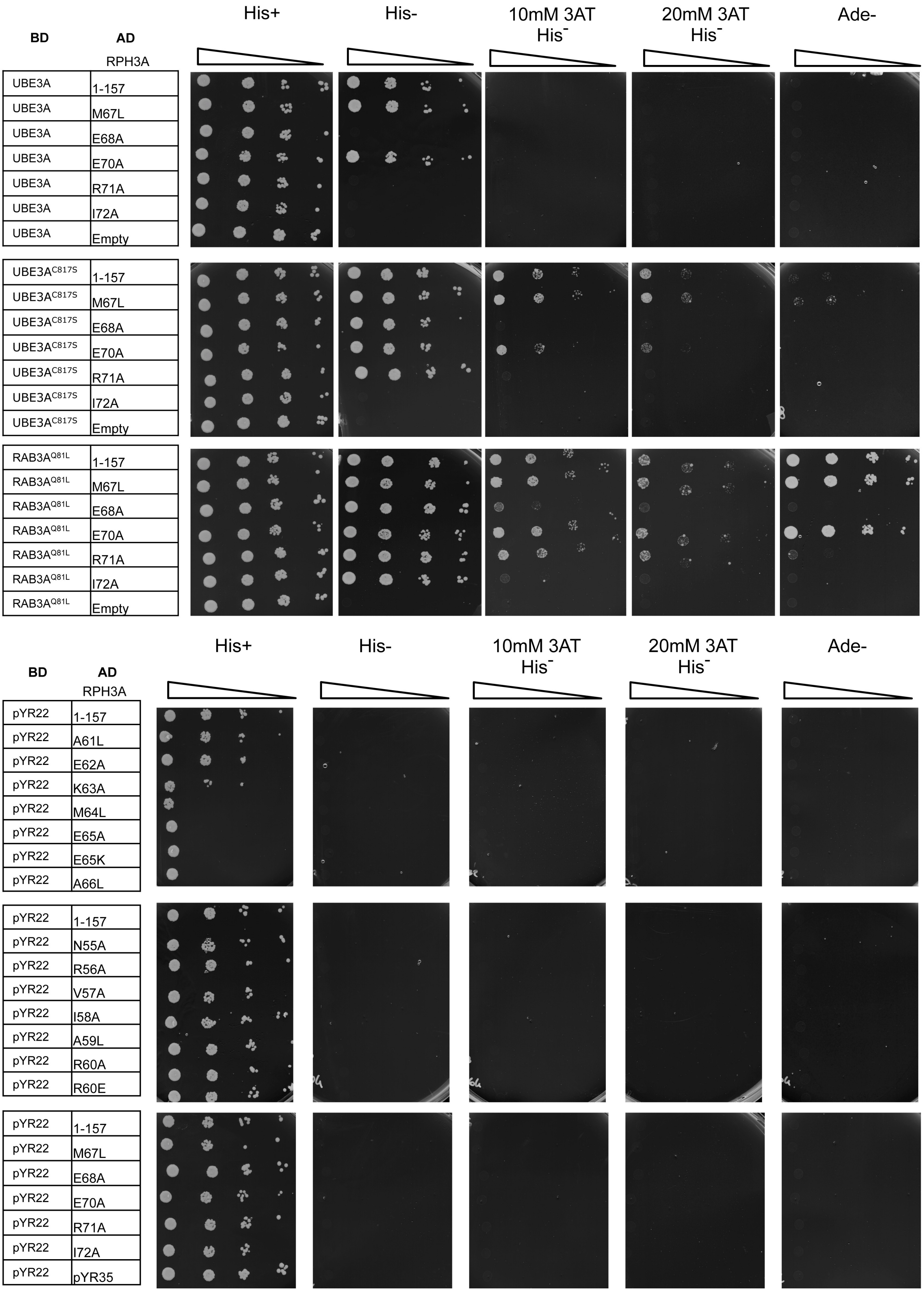

Supplementary Figure S4. Identification of the RPH3A residues involved in UBE3A and RAB3A interaction. Original plates of the spot assays corresponding to **Figure 3a**.

Supplementary Figure 5

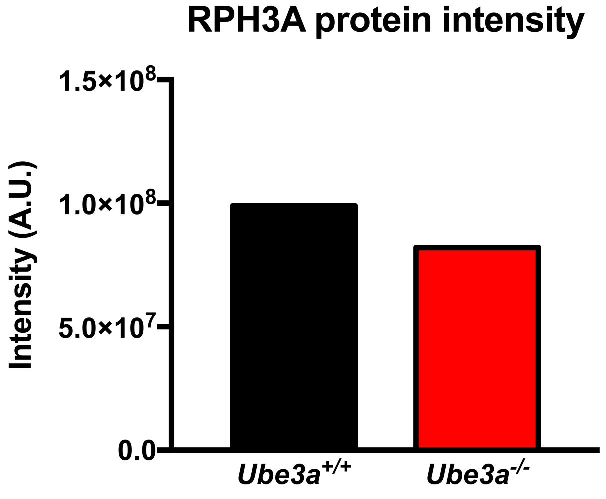

Supplementary Figure S5. RPH3A protein levels in *Ube3a*$^{m+/p+}$ and *Ube3a*$^{m-/p-}$ mice. RPH3A protein intensities in cortical brain lysates *Ube3a*$^{m+/p+}$ and *Ube3a*$^{m-/p-}$ mice as determined by mass spectrometry.

Supplementary Figure 6

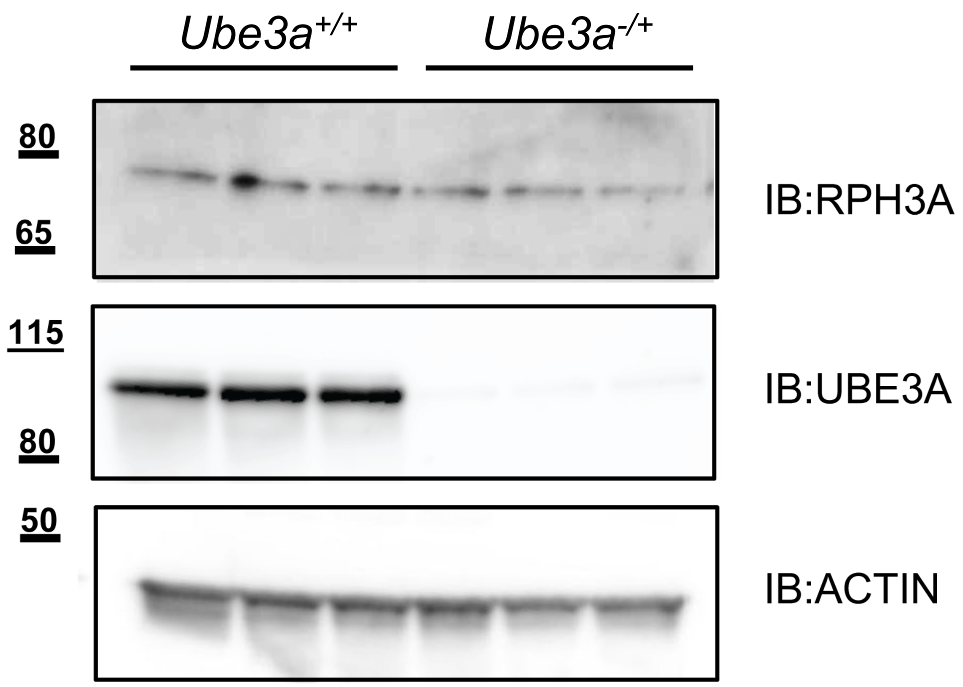

Supplementary Figure S6. RPH3A levels are not increased in AS mice. Cortical lysates obtained form WT (*Ube3a*$^{m+/p+}$) and AS (*Ube3a*$^{m-/p+}$) mice were analyzed by SDS-PAGE and immunoblotting using antibodies directed against RPH3A and UBE3A. ACTIN was used as loading control. Blot used for quantification as shown in **Figure 6k**.

Supplementary Figure 7

BD	E6	AD
UBE3A	-	RPH3A170
UBE3A	+	RPH3A170
C817S	-	RPH3A170
C817S	+	RPH3A170
UBE3A	-	p53
UBE3A	+	p53
C817S	-	p53
C817S	+	p53

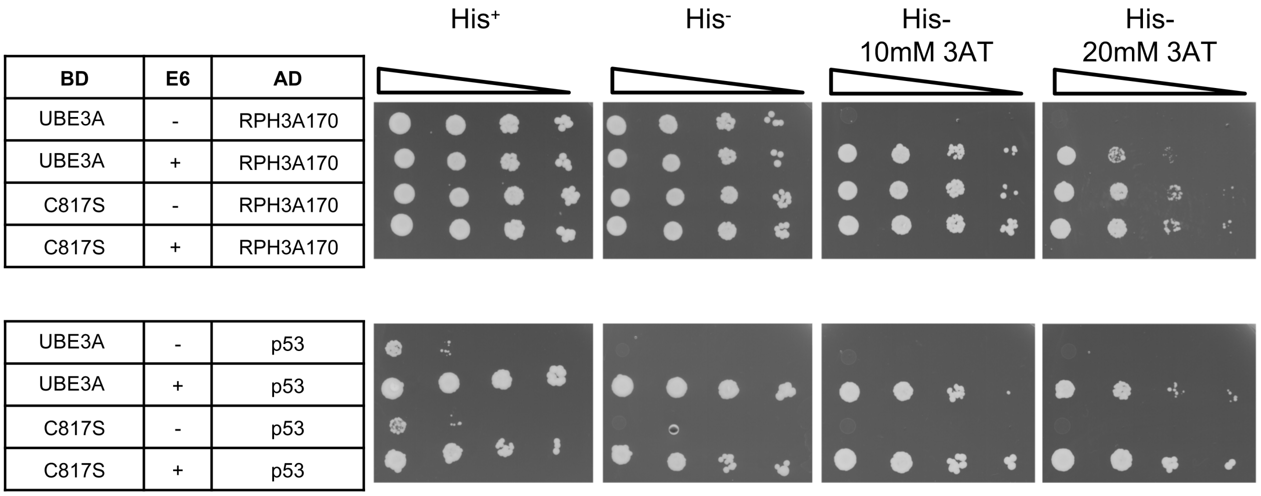

Supplementary Figure S7. E6 enhances the RPH3A-UBE3A interaction. *S. cerevisiae* strain PJ69a was co-transformed with UBE3A or UBE3A^{C817S} (baits) and RPH3A$^{1\text{-}170}$ or p53 (preys), and non-fused E6 or empty vector (pRA1). Cells were spotted on selective plates as indicated.

Supplementary Figure 8

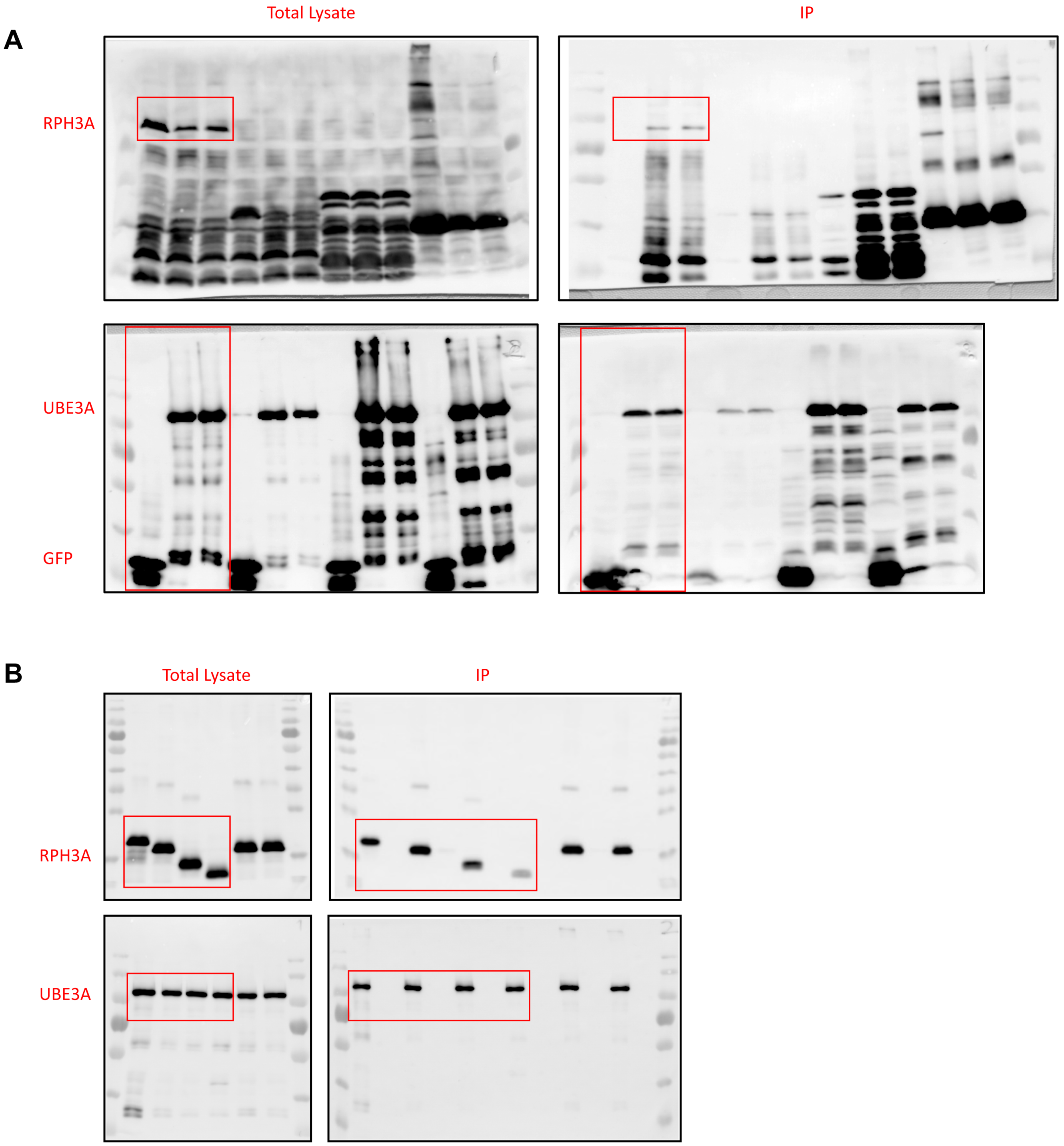

Supplementary Figure S8. Full-length blots. Full blots corresponding to **Figures 2a and b**

Supplementary Figure 9

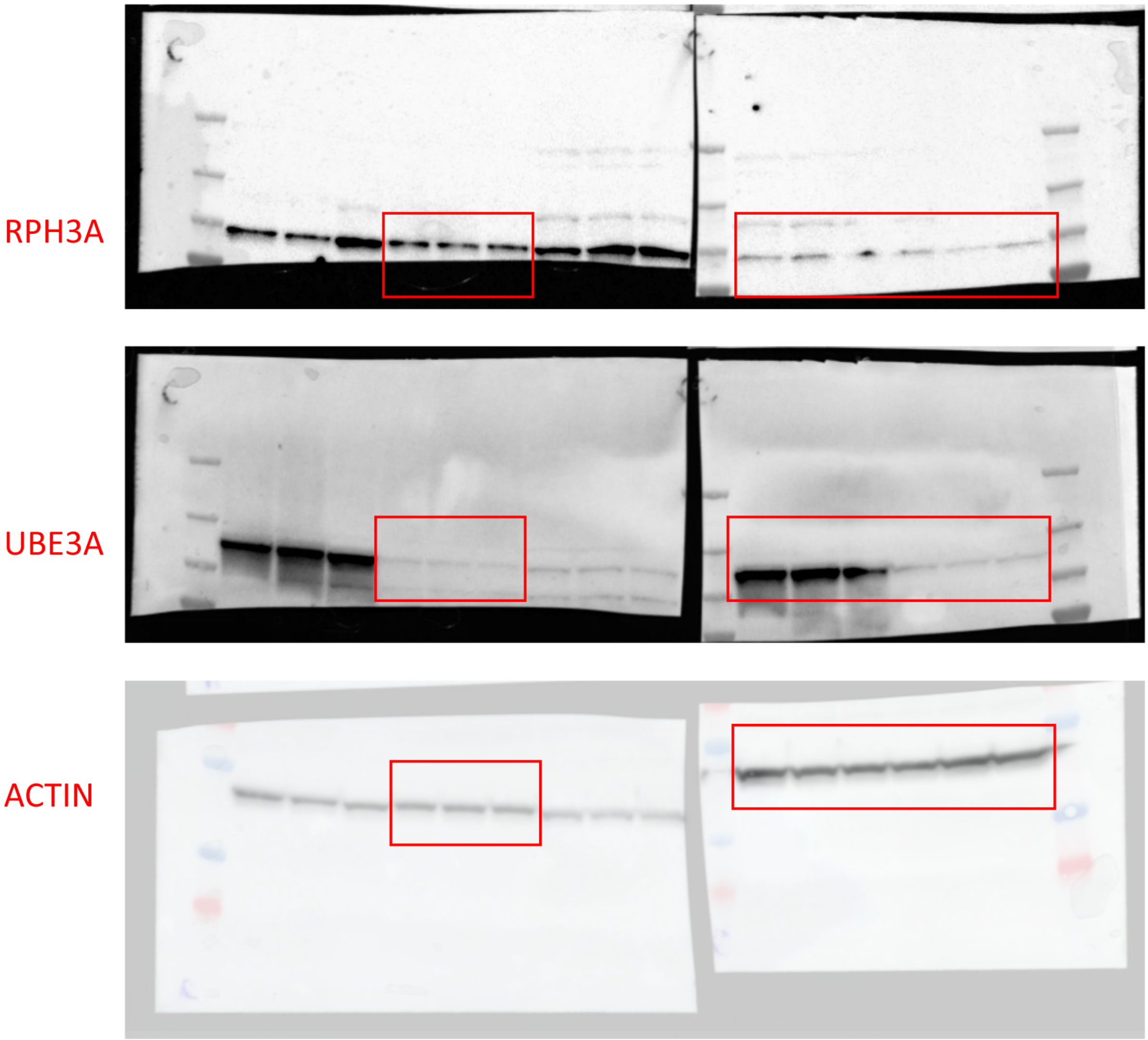

Supplementary Figure S9. Full-length blots. Full blots corresponding to **Figure 5a.** Blotted membranes were cut prior to antibody incubation.

Supplementary Figure 10

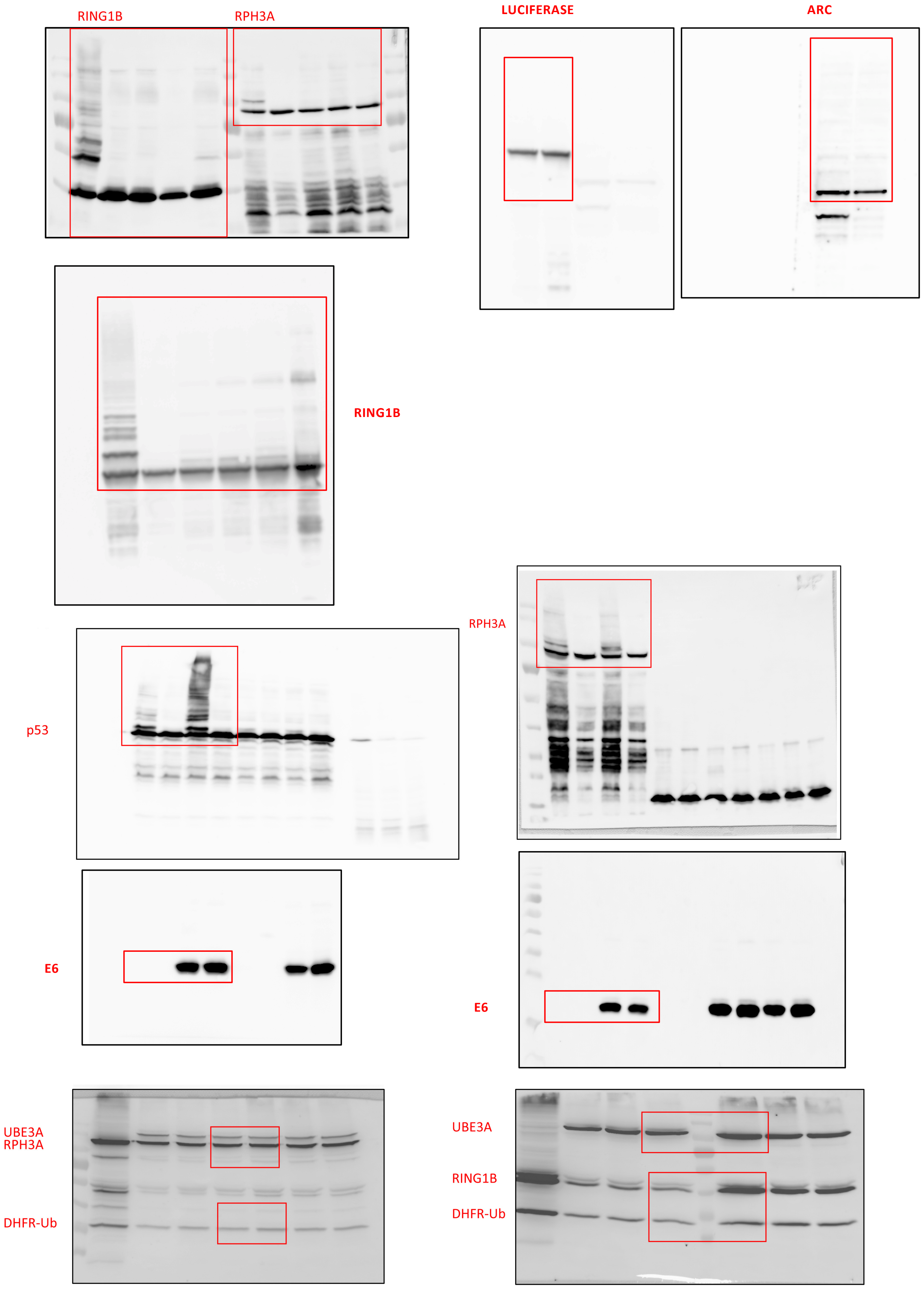

Supplementary Figure S10. Full-length blots. Full blots corresponding to **Figure 6d-j**

Supplementary Figure 11

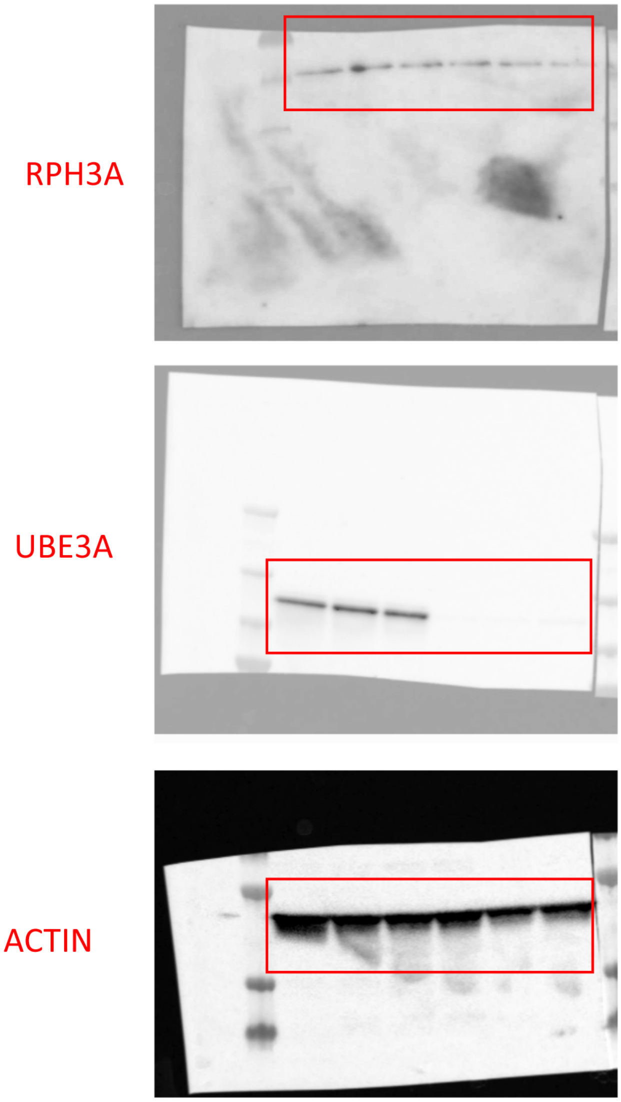

Supplementary Figure S11. Full-length blots. Full blots used for quantification in **Figure 6k** (see also **Supplementary Figure S6**).

Supplementary Figure 12

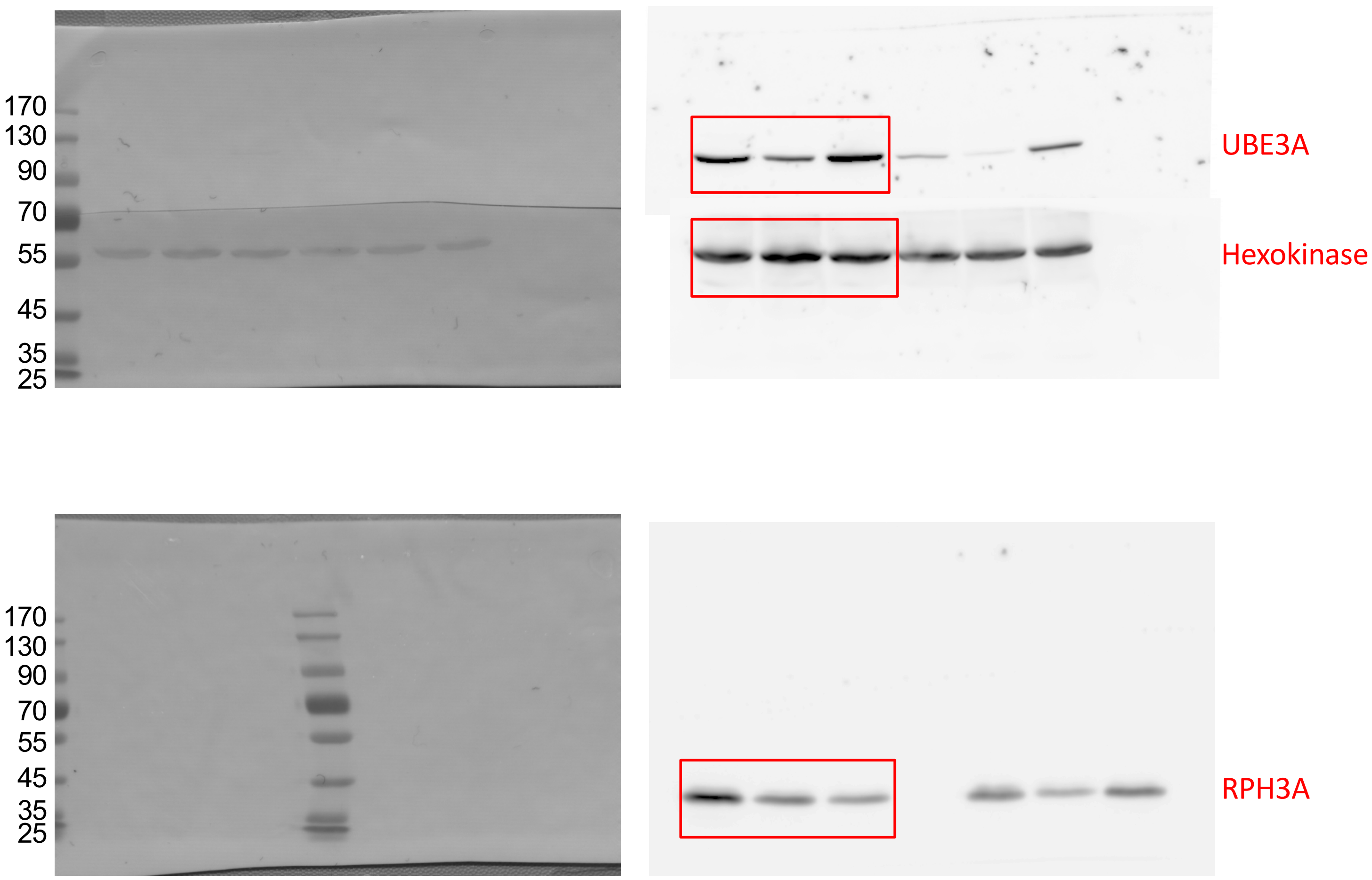

Supplementary Figure S12. Full-length blots. Full blots corresponding to **Figure S3b**.

Supplementary Table 1: Primers used in this study

Name	Sequence	Purpose
p881	GGCGCGCCTATGAAGCGAGCAGCTGCAAAGC	PCR UBE3A (Fw)
p882	GTCGACTTACCTAATCACAACAGATTC	PCR UBE3A (Rv)
RA80	AAGGATCCGACATGTCCGCCACAGACTCTCGCTATG	PCR RAB3A (Fw)
RA81	AAGCGGCCGCGTCGACTCAGCAGGCACAATCCTGATG	PCR RAB3A (Rv)
RA12	AAAGGCGCGCCTATGACTGACACTGTGGTGAAC	PCR RPH3A (Fw)
RA13	AAAGTCGACCTAATCACTGGACACGTGG	PCR RPH3A FL (Rv)
RA101	AAGCGGCCGCGTCGACTTAACCTTTGAAGAACCATGCTCC	PCR RPH3A 1-157 (Rv)
RA78	AAGCGGCCGCGTCGACTTACTTTATAGGCATGGGCTGTGG	PCR RPH3A 1-170 (Rv)
RA120	AAGGATCCGACATGTCTAGGCAGAGGAAGCAGGAAGAAC	PCR RPH3A 39-157 (Fw)
RA119	AAGGATCCGACATGTCTCGCATTGGGCGCCTGGTG	PCR RPH3A 71-157 (Fw)
RA99	AAGGATCCGACATGTCTGGAGATGGCGTGAACCGCT	PCR RPH3A 87-157 (Fw)
RA118	AAGCGGCCGCGTCGACTTAGCGCTTCCAGACCTCTCTCT	PCR RPH3A 1-149 (Rv)
RA121	AAGCGGCCGCGTCGACTTAAGCCACATTCTTCCTCATGGTC	PCR RPH3A 1-87 (Rv)
RA562	AAGGATCCGACATGTCTGAACTGACAGACGAGGAG	PCR RPH3A 45-87 (Fw)
RA102	CTGGAAGCGCTCAGGAGCAGCGGCAGCCAAAGGTTTCCCC AAGCAG	SDM RPH3A 1-157 SGAAAA (Fw)
RA103	CTGCTTGGGGAAACCTTTGGCTGCCGCTGCTCCTGAGCGC TTCCAG	SDM RPH3A 1-157 SGAAAA (Rv)
RA135	GCCAGAGTGATTGCTCGGGCAGAGAAGATGGAAGCCATGG AACAGGAACGCATTGGACGT	SDM RPH3A 1-157 N55A (Fw)
RA136	CCAATGCGTTCCTGTTCCATGGCTTCCATCTTCTCTGCCCG AGCAATCACTCTGGCGATG	SDM RPH3A 1-157 N55A (Rv)
RA137	AACGCAGTGATTGCTCGGGCAGAGAAGATGGAAGCCATGG AACAGGAACGCATTGGACGT	SDM RPH3A 1-157 R56A (Fw)
RA138	CCAATGCGTTCCTGTTCCATGGCTTCCATCTTCTCTGCCCG AGCAATCACTGCGTTGATG	SDM RPH3A 1-157 R56A (Rv)
RA139	AACAGAGCGATTGCTCGGGCAGAGAAGATGGAAGCCATGG AACAGGAACGCATTGGACGT	SDM RPH3A 1-157 V57A (Fw)
RA140	CCAATGCGTTCCTGTTCCATGGCTTCCATCTTCTCTGCCCG AGCAATCGCTCTGTTGATG	SDM RPH3A 1-157 V57A (Rv)
RA141	AACAGAGTGGCTGCTCGGGCAGAGAAGATGGAAGCCATGG AACAGGAACGCATTGGACGT	SDM RPH3A 1-157 I58A (Fw)
RA142	CCAATGCGTTCCTGTTCCATGGCTTCCATCTTCTCTGCCCG AGCAGCCACTCTGTTGATG	SDM RPH3A 1-157 I58A (Rv)
RA143	AACAGAGTGATTCTTCGGGCAGAGAAGATGGAAGCCATGGA ACAGGAACGCATTGGACGT	SDM RPH3A 1-157 A59L (Fw)
RA144	CCAATGCGTTCCTGTTCCATGGCTTCCATCTTCTCTGCCCG AAGAATCACTCTGTTGATG	SDM RPH3A 1-157 A59L (Rv)
RA166	AACAGAGTGATTGCTGCTGCAGAGAAGATGGAAGCCATGGA ACAGGAACGCATTGGACGT	SDM RPH3A 1-157 R60A (Fw)
RA167	CCAATGCGTTCCTGTTCCATGGCTTCCATCTTCTCTGCAGCA GCAATCACTCTGTTGATG	SDM RPH3A 1-157 R60A (Rv)
RA147	AACAGAGTGATTGCTGAGGCAGAGAAGATGGAAGCCATGG AACAGGAACGCATTGGACGT	SDM RPH3A 1-157 R60E (Fw)
RA148	CCAATGCGTTCCTGTTCCATGGCTTCCATCTTCTCTGCCTCA GCAATCACTCTGTTGATG	SDM RPH3A 1-157 R60E (Rv)
RA168	AACAGAGTGATTGCTCGGTTGGAGAAGATGGAAGCCATGGA ACAGGAACGCATTGGACGT	SDM RPH3A 1-157 A61L (Fw)

RA170	CCAATGCGTTCCTGTTCCATGGCTTCCATCTTCTCCAACCGAGCAATCACTCTGTTGATG	SDM RPH3A 1-157 A61L (Rv)
RA171	AACAGAGTGATTGCTCGGGCAGCTAAGATGGAAGCCATGGAACAGGAACGCATTGGACGT	SDM RPH3A 1-157 E62A (Fw)
RA172	CCAATGCGTTCCTGTTCCATGGCTTCCATCTTAGCTGCCCGAGCAATCACTCTGTTGATG	SDM RPH3A 1-157 E62A (Rv)
RA173	AACAGAGTGATTGCTCGGGCAGAGGCTATGGAAGCCATGGAACAGGAACGCATTGGACGT	SDM RPH3A 1-157 K63A (Fw)
RA174	CCAATGCGTTCCTGTTCCATGGCTTCCATAGCCTCTGCCCGAGCAATCACTCTGTTGATG	SDM RPH3A 1-157 K63A (Rv)
RA175	AACAGAGTGATTGCTCGGGCAGAGAAGTTGGAAGCCATGGAACAGGAACGCATTGGACGT	SDM RPH3A 1-157 M64A (Fw)
RA176	CCAATGCGTTCCTGTTCCATGGCTTCCAACTTCTCTGCCCGAGCAATCACTCTGTTGATG	SDM RPH3A 1-157 M64A (Rv)
RA149	AACAGAGTGATTGCTCGGGCAGAGAAGATGAAAGCCATGGAACAGGAACGCATTGGACGT	SDM RPH3A 1-157 E65K (Fw)
RA150	CCAATGCGTTCCTGTTCCATGGCTTTCATCTTCTCTGCCCGAGCAATCACTCTGTTGATG	SDM RPH3A 1-157 E65K (Rv)
RA177	AACAGAGTGATTGCTCGGGCAGAGAAGATGGCTGCCATGGAACAGGAACGCATTGGACGT	SDM RPH3A 1-157 E65A (Fw)
RA178	CCAATGCGTTCCTGTTCCATGGCAGCCATCTTCTCTGCCCGAGCAATCACTCTGTTGATG	SDM RPH3A 1-157 E65A (Rv)
RA179	AACAGAGTGATTGCTCGGGCAGAGAAGATGGAATTGATGGAACAGGAACGCATTGGACGT	SDM RPH3A 1-157 A66L (Fw)
RA180	CCAATGCGTTCCTGTTCCATCAATTCCATCTTCTCTGCCCGAGCAATCACTCTGTTGATG	SDM RPH3A 1-157 A66L (Rv)
RA181	AACAGAGTGATTGCTCGGGCAGAGAAGATGGAAGCCTTGGAACAGGAACGCATTGGACGT	SDM RPH3A 1-157 M67L (Fw)
RA182	CCAATGCGTTCCTGTTCCAAGGCTTCCATCTTCTCTGCCCGAGCAATCACTCTGTTGATG	SDM RPH3A 1-157 M67L (Rv)
RA183	AACAGAGTGATTGCTCGGGCAGAGAAGATGGAAGCCATGGCTCAGGAACGCATTGGACGT	SDM RPH3A 1-157 E68A (Fw)
RA184	CCAATGCGTTCCTGAGCCATGGCTTCCATCTTCTCTGCCCGAGCAATCACTCTGTTGATG	SDM RPH3A 1-157 E68A (Rv)
RA187	AACAGAGTGATTGCTCGGGCAGAGAAGATGGAAGCCATGGAACAGGCTCGCATTGGACGT	SDM RPH3A 1-157 E70A (Fw)
RA188	CCAATGCGAGCCTGTTCCATGGCTTCCATCTTCTCTGCCCGAGCAATCACTCTGTTGATG	SDM RPH3A 1-157 E70A (Rv)
RA189	AACAGAGTGATTGCTCGGGCAGAGAAGATGGAAGCCATGGAACAGGAAGCTATTGGACGT	SDM RPH3A 1-157 R71A (Fw)
RA190	CCAATAGCTTCCTGTTCCATGGCTTCCATCTTCTCTGCCCGAGCAATCACTCTGTTGATG	SDM RPH3A 1-157 R71A (Rv)
RA145	AACAGAGTGATTGCTCGGGCAGAGAAGATGGAAGCCATGGAACAGGAACGCGCTGGACGT	SDM RPH3A 1-157 I72A (Fw)
RA146	CCAGCGCGTTCCTGTTCCATGGCTTCCATCTTCTCTGCCCGAGCAATCACTCTGTTGATG	SDM RPH3A 1-157 I72A (Rv)
RA82	GGGACACAGCAGGGCTCGAGCGGTACCGCACC	SDM RAB3A Q81L (Fw)
RA83	GGTGCGGTACCGCTCGAGCCCTGCTGTGTCCC	SDM RAB3A Q81L (Rv)
MB3000	GGCCGCaaggagatatacaATGGCTGAACAAAAATTGATTTCTGAAGAGGATTTGTCTGGTGCTTCTgacgtcCTGCAGGctGTTTAAACttaattaaC	construction rbs-Myc-MCS (dsOligo Fw)
MB3001	ctagGttaattaaGTTTAAACagCCTGCAGgacgtcAGAAGCACCAGACAAATCCTCTTCAGAAATCAATTTTTGTTCAGCCATtgtatatctccttGC	construction rbs-Myc-MCS (dsOligo Rv)

MB3002	CCTGCAGGctATGtttcaggacccacaggagcg	PCR E6 type 16 (Fw)
MB3003	ttaattaaTTACAGCTGGGTTTCTCTACGTG	PCR E6 type 16 (Rv)
MB3004	AATTCgtTAAgAGGCCTgTAAgcTAAgg	deletion E2 (Fw)
MB3005	CGCGccTTAgcTTAcAGGCCTcTTAacG	deletion E2 Rv)
MB3006	CGCGCCtTAAggTAAgcTAAgacgt	deletion E1 (Fw)
MB3007	cTTAgcTTAccTTAaGG	deletion E1 (Rv)
MB3008	CTAGAAATAATTTTGTTTAACTTTAAG	deletion Ub (Fw)
MB3009	aattCTTAAAGTTAAACAAAATTATTT	deletion Ub (Rv)
p5098	GGCGCGCCGATGTGCAATACCAACATGTCTGTACCTAC	cloning MDM2 (Fw)
p5099	AATTAAGTGCGGCCGCTCGACCCG	cloning MDM2 (Rv)
p3991	GGCGCGCCTGTGGAAGACGCCAAAAACATAAAGAAA	cloning Luciferase (Fw)
p3992	GCGGCCGCTTACACGGCGATCTTTC	cloning Luciferase (Rv)
p5131	CGCGCCAACGTCTGGCGCGGGTAC	cloning NEDD4 (Fw)
p5132	CCGCGCCAGACGTTGG	cloning NEDD4 (Rv)
RA220	CTCAATTTGTTCATTATTGTAATGGGGAATAGTAATCTCCAC AGTCCTGAATATC	SDM E269G (Fw)
RA221	GATATTCAGGACTGTGGAGATTACTATTCCCCATTACAAT AATGAACAAATTGAG	SDM E269G (Rv)
RA224	CATTATTGTAATGGAGAATAGTAATTTCCACAGTCCTGAA TATCTGGAAATG	SDM L273F (Fw)
RA225	CATTTCCAGATATTCAGGACTGTGGAAATTACTATTCTC CAT TACAATAATG	SDM L273F (Rv)

Supplementary Table 2: Plasmids generated and used in this study

Name	Insert	Purpose
pYR22	Empty	Y2H
pGADT7	Empty	Y2H
pYR35	Empty	Y2H
pRA55	UBE3A WT	Y2H
pRA61	UBE3A C817S	Y2H
pRA72	RAB3A Q81L	Y2H
pRA28	RPH3A FL	Y2H
pRA77	RPH3A 1-157	Y2H
pRA87	RPH3A 1-157 SGAAAA	Y2H
pRA98	RPH3A 39-157	Y2H
pRA99	RPH3A 71-157	Y2H
pRA89	RPH3A 87-149	Y2H
pRA86	RPH3A 1-149	Y2H
pRA100	RPH3A 1-87	Y2H
pRA106	RPH3A 39-87	Y2H
pRA110	RPH3A 45-87	Y2H
pMB277	E1, UbcH7, Ub wt	*E. coli* expression
pMB278	E1, UbcH7, ΔUb	*E. coli* expression
pMB280	V5-PEX22, HA-GFP	*E. coli* expression
pMB284	V5-RPH3A FL, HA-GFP	*E. coli* expression
pMB286	V5-RPH3A FL, HA-UBE3A WT	*E. coli* expression
pMB287	V5-RPH3A FL, HA-UBE3A C817S	*E. coli* expression
pMB316	E1, ΔE2, Ub	*E. coli* expression
pMB318	ΔE1, UbcH7, Ub	*E. coli* expression
pMB323	V5-PEX22, HA-GFP, Myc/MCS	*E. coli* expression
pMB326	V5-RPH3A 1-157, HA-UBE3A WT	*E. coli* expression
pMB327	V5-RPH3A 39-157, HA-UBE3A WT	*E. coli* expression
pMB328	V5-RPH3A 71-157, HA-UBE3A WT	*E. coli* expression
pMB336	V5-PEX22, HA-GFP, Myc-E6	*E. coli* expression
pMB338	V5-RPH3A FL, HA-UBE3A WT, Myc-E6	*E. coli* expression
pMB344	V5-p53, HA-UBE3A WT	*E. coli* expression
pMB345	V5-p53, HA-UBE3A C817S	*E. coli* expression
pMB346	V5-p53, HA-UBE3A WT, Myc-E6	*E. coli* expression
pMB347	V5-p53, HA-UBE3A C817S, Myc-E6	*E. coli* expression
pMB419	V5/MCS	*E. coli* expression
pMB427	V5-RPH3A FL	*E. coli* expression
pYW5	HA/MCS-Myc/MCS	*E. coli* expression
pMB436	HA-UBE3A WT	*E. coli* expression
pMB437	HA-UBE3A C817S	*E. coli* expression
pMB443	V5-RING1B I53S	*E. coli* expression
pRA172	RPH3A 1-157 N55A	Y2H
pRA173	RPH3A 1-157 R56A	Y2H

pRA174	RPH3A 1-157 V57A	Y2H
pRA175	RPH3A 1-157 I58A	Y2H
pRA176	RPH3A 1-157 A59L	Y2H
pRA141	RPH3A 1-157 R60A	Y2H
pRA177	RPH3A 1-157 R60E	Y2H
pRA131	RPH3A 1-157 A61L	Y2H
pRA142	RPH3A 1-157 E62A	Y2H
pRA143	RPH3A 1-157 K63A	Y2H
pRA132	RPH3A 1-157 M64A	Y2H
pRA178	RPH3A 1-157 E65K	Y2H
pRA133	RPH3A 1-157 E65A	Y2H
pRA134	RPH3A 1-157 A66L	Y2H
pRA135	RPH3A 1-157 M67L	Y2H
pRA136	RPH3A 1-157 E68A	Y2H
pRA137	RPH3A 1-157 E70A	Y2H
pRA138	RPH3A 1-157 R71A	Y2H
pRA179	RPH3A 1-157 I72A	Y2H
pRA158	UBE3A E269G	Y2H
pRA159	UBE3A L273F	Y2H
pRA1	Empty	Y3H
pRA117	RAB3A Q81L	Y3H
pRA125	RPH3a 1-170	Y3H
pRA126	RPH3A 1-157 SGAAAA	Y3H
pRA53	UBE3A WT	*S. cerevisiae* expression
pRA62	UBE3A C817S	*S. cerevisiae* expression
pRA34	RPH3A FL	*S. cerevisiae* expression
pRA64	UBE3A WT	HEK293T expression
pRA65	UBE3A C817S	HEK293T expression
pRA66	RPH3A FL	HEK293T expression
pMS1	RING1B I53S	HEK293T expression
pYE2009	V5-ARC	*E. coli* expression
pYE2010	V5-Luciferase (firefly)	*E. coli* expression
pYE2011	HA-MDM2 (human)	*E. coli* expression
pYE2012	HA-NEDD4 (human)	*E. coli* expression
